# Source of the Catamenial Discharge

**Published:** 1847-07

**Authors:** George King


*Source of the Catamenial Discharge.*—By George King, M. D.—The physiology of the source of the catamenial discharge, so peculiar to the human female, and the functions of the interior of the uterus positively ascertained, are subjects comparatively of very recent discovery, and as the opportunities are so rare of our having proof or of our obtaining any decisive means of determining the matter or establishing the fact that the uterus is the source of this healthy and proper sexual secretion, I think the following evidence may be interesting to the readers of the *Provincial Medical and Surgical Journal.* Should you be of that opinion perhaps you will find room for it in an early number :—

On Saturday, the 27th ult., I was applied to by a medical friend to assist him at a *post-mortem* examination, under an order from the city coroner, of a woman who had hung herself, the jury not being able to agree in their verdict. It is not often that a medical man is called on to make a *post-mortem* examination for the purpose of assisting the jury in coming to a correct verdict after death from hanging, the cause of death being so palpably visible ; and how those twelve wiseacres could suppose that we should, by an examination after death, be able to discover the motive that could have induced this poor creature to commit such a rash act, I cannot conceive. But their ignorance was bliss *to us*, as it gave us an opportunity of making a very interesting examination.

The external appearance, and the gorged state of the blood-vessels of the brain, clearly proved that death had been caused by strangulation, and it also proved Dr. G. Burrows' theory on this subject to be correct. In removng the abdominal viscera we were struck with the size and vascular appearance of the uterus. As we understood she had been confined three months before, and the child soon after died, we thought the uterus might be impregnated, but on laying it open it presented to our view a most beautiful velvet-like appearance; the whole internal surface was covered with a dark, sanguineous mucus, which seemed to be exudating from it and could be easily scraped off. This unusual appearance we at once suspected to be the catamenial secretion, or the commencement of the process of menstruation.—There was no appearance of any discharge in the vagina, and in order to satisfy ourselves on the point as to whether she had been regular since the birth of the last child, we made inquiry, and learnt from a female friend who lived in the house with her, that she had menstruated *once* since her confinement, and she thought that she was expecting it again in a day or two. There is then indisputable evidence, and the strongest corroborative proof of the fact, that the source of the menstrual discharge, once so much disputed, is the inner membrane lining the uterus, and I think the strongest case recorded. As it is well known, and many remarkable cases are recorded, that hanging has a very curious effect on the organs of generation of the male,—Query, Did the apparently enlarged uterus, and the vascularity of the external part of this organ, arise from the process going on within, or from the mode of death? Perhaps some of your learned readers may be able to inform me.—*Prov. Med. & Surg. Journal.*

